# Fasting status modifies the association between triglyceride and all‐cause mortality: A cohort study

**DOI:** 10.1002/hsr2.642

**Published:** 2022-05-16

**Authors:** Yan Fang, Yutang Wang

**Affiliations:** ^1^ Discipline of Life Science, School of Science, Psychology, and Sport Federation University Australia Ballarat Victoria Australia

**Keywords:** biomarker, fasting, mortality, nonfasting, triglyceride

## Abstract

**Background and Aims:**

Both fasting and non‐fasting levels of triglyceride have been shown positively associated with all‐cause mortality. It is unknown whether fasting status modifies this association. This study aimed to address this question.

**Methods:**

This study included 34,512 US adults (27,036 fasting and 7476 nonfasting participants). All‐cause mortality was ascertained by linkage to the National Death Index records. Cox proportional hazards models were used to estimate hazard ratios of triglyceride for mortality.

**Results:**

This cohort was followed up for a mean of 13.0 years. During the follow‐up, 8491 all‐cause deaths were recorded. A 1‐natural‐log‐unit increase in triglyceride was associated with an 8% higher multivariate‐adjusted risk of all‐cause mortality. Interaction analyses showed that fasting status interacted with triglyceride in predicting all‐cause mortality. Sub‐analyses showed that a 1‐natural‐log‐unit increase in triglyceride was associated with a 17% higher multivariate‐adjusted risk of all‐cause mortality in the nonfasting subcohort; however, there lacked such an association in the fasting sub‐cohort. Similarly, high (200–499 mg/dL) and very high levels of triglyceride (≥500 mg/dL) were associated with higher all‐cause mortality risks compared with low normal triglyceride (<100 mg/dL) only in the nonfasting subcohort.

**Conclusion:**

This study found that, compared to fasting triglyceride, nonfasting triglyceride was more sensitive in predicting all‐cause mortality. This study supports the initiatives by some guidelines to recommend the use of nonfasting triglycerides for risk assessment.

## 1 INTRODUCTION

The association between triglyceride and all‐cause mortality is still under debate. Some studies showed that fasting triglyceride was positively associated with all‐cause mortality,^1^, ^2^, ^3^ whereas others failed to find such an association.^4^, ^5^ The situation is similar for non‐fasting triglyceride: nonfasting triglyceride has been found associated^6^, ^7^ or not associated with all‐cause mortality.^8^ Recently, it was reported that triglyceride was also positively associated with all‐cause mortality when samples with mixed fasting status were used (i.e., some samples were from fasting participants whereas the others were from nonfasting participants).^9^, ^10^

However, it is unknown whether fasting status modifies the association between triglyceride and all‐cause mortality. In other words, it is unknown whether fasting triglyceride is better in predicting all‐cause mortality than nonfasting triglyceride, or vice versa. This study aimed to address this question using US adults who attended the National Health and Nutrition Examination Survey (NHANES) from 1988 to 2014.

## 2 METHODS

### 2.1 Study participants

The design of the study mainly followed a previously published report.^3^ This cohort study included participants from NHANES III (1988–1994) and the subsequent eight cycles of NHANES from 1999 to 2014.^11^, ^12^, ^13^ The inclusion criteria included age of ≥20 years and presence of triglyceride data. This resulted in a cohort of 35,035 participants. The following participants were excluded: those without a follow‐up time or with a follow‐up time of 0 month (*N* = 77), and those without a fasting time (*N* = 329) or with a fasting time ≥24 h (*N* = 117). Therefore, a total of 34,512 participants were included in the final analysis.

### 2.2 Ethics considerations

The National Center for Health Statistics Research Ethics Review Board (ERB) approved all study protocols (ERB Numbers: NHANES III, NHANES Protocol #98–12, NHANES Protocol #2005–06, and NHANES Protocol #2011–17). All procedures were performed following the guidelines of the Declaration of Helsinki. Written informed consent was obtained from all participants. The participants' records were anonymized before being accessed by the authors.^11^, ^12^, ^13^

### 2.3 Definition of fasting status

Fasting status was defined as a fasting time of ≥8 h^14^, ^15^ and nonfasting status as a fasting time of <8 h.

### 2.4 Triglyceride classification

The baseline concentration of triglyceride in the serum was directly retrieved from the NHANES website.^11^, ^12^, ^13^ The participants were divided into five groups according to triglycerides levels as previously reported,^3^ that is, low‐normal (<100 mg/dL), high‐normal (100–149 mg/dL), borderline‐high (150–199 mg/dL), high (200–499 mg/dL), and very high (≥500 mg/dL). This classification was based on the four categories (normal, borderline‐high, high, and very high) recommended by the National Cholesterol Education Program (NCEP) Expert Panel,^16^ with further categorizing the normal triglyceride levels into low‐normal and high‐normal as previously reported.^3^

### 2.5 Mortality ascertainment

Data on all‐cause mortality were directly retrieved from NHANES‐linked mortality files.^11^, ^12^, ^13^ To evaluate mortality status, the National Center for Health Statistics conducted probabilistic matching to link the NHANES data with death certificate records from the National Death Index (NDI) records. Follow‐up time was defined as the time (in months) from the time when the blood was drawn at the Mobile Examination Center until death, or until the end of follow‐up (i.e., December 31, 2015), whichever occurred first.^11^, ^12^, ^13^

### 2.6 Covariates

Confounding covariates were similar to the previous publications^11^, ^12^, ^13^, ^17^, ^18^ and included age (continuous), sex (male or female), ethnicity (Non‐Hispanic White, Non‐Hispanic Black, Mexican‐American, or other), obesity (underweight, normal, overweight, obese, or unknown), poverty‐income ratio (<130%, 130%–349%, ≥350%, or unknown), education (<high school, high school, >high school, or unknown), and survey periods (1988–1991, 1991–1994, 1999–2000, 2001–2002, 2003–2004, 2005–2006, 2007–2008, 2009–2010, 2011–2012, or 2013–2014). Lifestyle confounders included physical activity (inactive, insufficiently active, active, or unknown), alcohol consumption (never, <1 drink per week, 1–6 drinks per week, ≥7 drinks per week, or unknown), and smoking status (past smoker, current smoker, nonsmoker, or unknown). Clinical confounders included self‐reported physician diagnosis of hypertension (yes, no, or unknown), hypercholesterolemia (yes or no), and diabetes (yes or no).

### 2.7 Statistical analyses

Data were presented as mean and standard deviation for normally distributed continuous variables, median and interquartile range for not normally distributed continuous variables or number and percentages for categorical variables.^19^ The difference in age among groups was analyzed using one‐way analysis of variance (ANOVA) and the difference in triglyceride (non‐normally distributed) among groups was analyzed using Kruskal–Wallis one‐way ANOVA.^20^ Differences among categorical variables were analyzed using Pearson's *χ* ^2^ test. Cox proportional hazards models^21^ were used to calculate hazard ratios (HRs) and 95% confidence intervals (CIs) of triglyceride for mortality, with or without adjustment for confounding factors including age, sex, ethnicity, obesity, education, poverty‐income ratio, lifestyle factors (physical activity, alcohol consumption, and smoking status), survey period, and clinical confounders (hypercholesterolemia, hypertension, and diabetes). Triglyceride was treated as a continuous variable (natural log‐transformed) or categorical variable in the models. Sub‐analyses were conducted in the fasting and nonfasting subcohorts and in the male and female subcohorts. Sensitivity analyses were conducted by further adjustment for history of heart attack (yes, no, or unknown) and stroke (yes, no, or unknown).

The null hypothesis was rejected for a two‐sided *p* value of <0.05. All analyses were performed using SPSS version 27.0 (IBM SPSS Statistics for Windows, IBM Corporation).

## 3 RESULTS

### 3.1 General characteristics

This study included 34,512 US adults (27,036 fasting and 7476 non‐fasting participants) with a mean (standard deviation) age of 49 (19) years. Baseline characteristics of the participants are described in Tables 1 and S1–S2. People with higher triglyceride had a higher percentage of males, had less income and education, and had a higher prevalence of hypercholesterolemia and diabetes in the whole cohort (Table 1) as well as in the fasting and non‐fasting subcohorts (Tables S1–S2).

**Table 1:** Baseline characteristics of the whole cohort of 34,512 adult participants

	Triglyceride concentration (mg/dL)						*p* for trend
	<100	100–149	150–199	200–499	≥500	Overall	
Sample size	14,173	9411	4992	5426	510	34,512	NA
Triglyceride, mg/dL, median (IQR)	73 (59–86)	121 (109–134)	170 (159–183)	253 (221–309)	626 (550–803)	113 (79–168)	<0.001
Age, y, mean (SD)	45 (19)	51 (19)	53 (18)	52 (18)	49 (15)	49 (19)	<0.001
Sex (male), *n* (%)	6229 (43.9)	4488 (47.7)	2456 (49.2)	2917 (53.8)	353 (69.2)	16,443 (47.6)	<0.001
Ethnicity, *n* (%)							<0.001
Non‐Hispanic White	5789 (40.8)	4353 (46.3)	2426 (48.6)	2676 (49.3)	236 (46.3)	15,480 (44.9)	
Non‐Hispanic Black	4501 (31.8)	1901 (20.2)	774 (15.5)	634 (11.7)	54 (10.6)	7864 (22.8)	
Mexican American	2461 (17.4)	2209 (23.5)	1299 (26.0)	1618 (29.8)	178 (34.9)	7765 (22.5)	
Other	1422 (10.0)	948 (10.1)	493 (9.9)	498 (9.2)	42 (8.2)	3403 (9.9)	
Obesity, *n* (%)							<0.001
Underweight	440 (3.1)	125 (1.3)	27 (0.5)	22 (0.4)	1 (0.2)	615 (1.8)	
Normal	6381 (45.0)	2805 (29.8)	1076 (21.6)	911 (16.8)	59 (11.6)	11,232 (32.5)	
Overweight	4196 (29.6)	3379 (35.9)	1915 (38.4)	2143 (39.5)	228 (44.7)	11,861 (34.4)	
Obese	3036 (21.4)	2998 (31.9)	1918 (38.4)	2282 (42.1)	219 (42.9)	10,453 (30.3)	
Unknown	120 (0.8)	104 (1.1)	56 (1.1)	68 (1.3)	3 (0.6)	351 (1.0)	
Poverty‐income ratio, *n* (%)							<0.001
<130%	3969 (28.0)	2663 (28.3)	1473 (29.5)	1697 (31.3)	176 (34.5)	9978 (28.9)	
130%–349%	5242 (37.0)	3597 (38.2)	1833 (36.7)	1999 (36.8)	204 (40.0)	12,875 (37.3)	
≥350%	3768 (26.6)	2386 (25.4)	1201 (24.1)	1241 (22.9)	91 (17.8)	8687 (25.2)	
Unknown	1194 (8.4)	765 (8.1)	485 (9.7)	489 (9.0)	39 (7.6)	2972 (8.6)	
Education, %							<0.001
<High School	4084 (28.8)	3262 (34.7)	1966 (39.4)	2216 (40.8)	217 (42.5)	11,745 (34.0)	
High School	3729 (26.3)	2470 (26.2)	1305 (26.1)	1425 (26.3)	143 (28.0)	9072 (26.3)	
>High School	6307 (44.5)	3641 (38.7)	1698 (34.0)	1770 (32.6)	149 (29.2)	13,565 (39.3)	
Unknown	53 (0.4)	38 (0.4)	23 (0.5)	15 (0.3)	1 (0.2)	130 (0.4)	
Physical activity, *n* (%)							<0.001
Inactive	4372 (30.8)	2456 (26.1)	1249 (25.0)	1325 (24.4)	144 (28.2)	9546 (27.7)	
Insufficiently active	5265 (37.1)	3594 (38.2)	1823 (36.5)	2045 (37.7)	183 (35.9)	12,910 (37.4)	
Active	4532 (32.0)	3357 (35.7)	1918 (38.4)	2055 (37.9)	183 (35.9)	12,045 (34.9)	
Alcohol consumption, *n* (%)							<0.001
0 drink/week	2299 (16.2)	1755 (18.6)	966 (19.4)	1096 (20.2)	97 (19.0)	6213 (18.0)	
<1 drink/week	2931 (20.7)	1948 (20.7)	1019 (20.4)	1020 (18.8)	70 (13.7)	6988 (20.2)	
1–6 drinks/week	3131 (22.1)	1789 (19.0)	856 (17.1)	897 (16.5)	93 (18.2)	6766 (19.6)	
≥7 drinks/week	1802 (12.7)	1182 (12.6)	620 (12.4)	686 (12.6)	91 (17.8)	4381 (12.7)	
Unknown	4010 (28.3)	2737 (29.1)	1531 (30.7)	1727 (31.8)	159 (31.2)	10,164 (29.5)	
Smoking status, *n* (%)							<0.001
Past smoker	3191 (22.5)	2161 (23.0)	1204 (24.1)	1315 (24.2)	150 (29.4)	8021 (23.2)	
Current smoker	2984 (21.1)	2469 (26.2)	1463 (29.3)	1638 (30.2)	161 (31.6)	8715 (25.3)	
Nonsmoker	7989 (56.4)	4776 (50.7)	2322 (46.5)	2471 (45.5)	199 (39.0)	17757 (51.5)	
Hypertension, *n* (%)	3421 (24.1)	3204 (34.0)	1827 (36.6)	2162 (39.8)	208 (40.8)	10,822 (31.4)	<0.001
Hypercholesterolemia, *n* (%)	2327 (16.4)	2469 (26.2)	1559 (31.2)	1940 (35.8)	230 (45.1)	8525 (24.7)	<0.001
Diabetes, *n* (%)	841 (5.9)	912 (9.7)	623 (12.5)	898 (16.5)	131 (25.7)	3405 (9.9)	<0.001

Abbreviations: IQR, interquartile range; NA, not applicable; SD, standard deviation.

### 3.2 Association of circulating triglyceride with all‐cause mortality

This cohort was followed up for 450,333 person‐years (mean follow‐up, 13.0 years). During the follow‐up, 8491 all‐cause deaths were recorded. A 1‐natural‐log‐unit increase in triglyceride was associated with an 8% higher multivariate‐adjusted risk of all‐cause mortality (HR, 1.08; 95% CI, 1.04–1.13, Table 2). The interaction analysis showed that triglyceride interacted with fasting status in predicting all‐cause mortality (Table 3). Subanalyses showed that, in the non‐fasting sub‐cohort, a 1‐natural‐log‐unit increase in triglyceride was associated with a 17% higher multivariate‐adjusted risk of all‐cause mortality (HR, 1.17; 95% CI, 1.09–1.25, Table 2), whereas there lacked such an association in the fasting subcohort (Table 2). In addition, the interaction analysis showed that triglyceride interacted with sex in predicting all‐cause mortality (Table S3): the positive association between triglyceride and all‐cause mortality presented in females but not in males (Table S4). Ethnicity did not interact with triglyceride in predicting all‐cause mortality (Table S5).

**Table 2:** Natural log‐transformed triglyceride and risk for all‐cause mortality among 34,512 adults

	Whole cohort (*N* = 34,512)			Fasting subcohort (*N* = 27,036)			Nonfasting subcohort (*N* = 7476)		
	HR	95% CI	*p* value	HR	95% CI	*p* value	HR	95% CI	*p* value
Model 1	1.51	1.46–1.56	<0.001	1.47	1.41–1.53	<0.001	1.53	1.44–1.62	<0.001
Model 2	1.15	1.10–1.20	<0.001	1.10	1.05–1.16	<0.001	1.21	1.13–1.30	<0.001
Model 3	1.12	1.08–1.17	<0.001	1.07	1.01–1.12	0.02	1.23	1.15–1.32	<0.001
Model 4	1.08	1.04–1.13	<0.001	1.05	0.99–1.10	0.11	1.17	1.09–1.25	<0.001

*Note*: Model 1: Unadjusted; Model 2: Adjusted for age, sex, and ethnicity; Model 3: Adjusted for all the factors in Model 2 plus obesity, poverty‐income ratio, education, physical activity, alcohol consumption, smoking status, and survey period; Model 4: Adjusted for all the factors in Model 3 plus hypercholesterolemia, hypertension, and diabetes.

Abbreviations: CI, confidence interval; HR, hazard ratio.

**Table 3:** Interaction of triglyceride with fasting status in predicting all‐cause mortality analyzed by Cox proportional hazards models

	HR^a^	95% CI	*p* value
LnTG X fasting status^b^	1.11	1.03–1.21	0.01

Abbreviations: CI, confidence interval; HR, hazard ratio; LnTG, natural log‐transformed triglyceride.

^a^ Adjusted for triglyceride (natural log‐transformed), fasting status, age, sex, ethnicity, obesity, poverty‐income ratio, education, physical activity, alcohol consumption, smoking status, survey period, hypercholesterolemia, hypertension, and diabetes.

^b^ The interaction factor, computed as natural log‐transformed triglyceride multiplied by fasting status (fasting and non‐fasting, coded as 0 and 1, respectively). The resulting interaction factor was treated as a continuous variable in the interaction analysis.

When triglyceride was treated as a categorical variable, people who had high triglyceride (HR, 1.09; 95% CI, 1.02–1.16) or very high triglyceride (HR, 1.19; 95% CI, 1.01–1.39) had higher risks of all‐cause mortality compared with those with low‐normal triglyceride (Table 4). Subanalyses showed that triglyceride was positively associated with all‐cause mortality risks in a dose‐dependent manner in the non‐fasting sub‐cohort, whereas there lacked such an association in the fasting subcohort (Table 4).

**Table 4:** Triglyceride (categorical) and risk for all‐cause mortality among 34,512 adults

Triglyceride (mg/dL)	No. of participants	HR^a^ (95% CI)	*p* value
Whole cohort (*N* = 34,512)			
<100 (low‐normal)	14,173	1 [reference]	NA
100–149 (high‐normal)	9411	1.01 (0.95–1.07)	0.76
150–199 (borderline‐high)	4992	1.04 (0.97–1.11)	0.27
200–499 (high)	5426	1.09 (1.02–1.16)	0.008
≥500 (very high)	510	1.19 (1.01–1.39)	0.04
Fasting sub‐cohort (*N* = 27,036)			
<100 (low‐normal)	11,519	1 [reference]	NA
100–149 (high‐normal)	7444	0.97 (0.91–1.04)	0.34
150–199 (borderline‐high)	3819	1.00 (0.92–1.08)	>0.99
200–499 (high)	3926	1.03 (0.95–1.12)	0.47
≥500 (very high)	328	1.08 (0.87–1.35)	0.47
Nonfasting subcohort (*N* = 7476)			
<100 (low‐normal)	2654	1 [reference]	NA
100–149 (high‐normal)	1967	1.12 (1.01–1.25)	0.03
150–199 (borderline‐high)	1173	1.14 (1.01–1.28)	0.03
200–499 (high)	1500	1.22 (1.09–1.37)	<0.001
≥500 (very high)	182	1.45 (1.15–1.83)	0.002

Abbreviations: CI, confidence interval; HR, hazard ratio; NA, not applicable; No., number.

^a^ Adjusted for age, sex, ethnicity, obesity, poverty‐income ratio, education, physical activity, alcohol consumption, smoking status, survey period, hypercholesterolemia, hypertension, and diabetes.

### 3.3 Sensitivity analyses

Sensitivity analyses showed that further adjustment for history of heart attack and stroke did not materially change the results (Table S6).

## 4 DISCUSSION

Using a representative cohort of US adults, this study found that fasting status modified the association between triglyceride and all‐cause mortality: nonfasting triglyceride, but not fasting triglyceride, was positively associated with all‐cause mortality risks after adjustment for all the tested confounders.

This study suggests that non‐fasting triglyceride may be better in predicting all‐cause mortality than its fasting counterpart. The results of this study are of clinical importance given that non‐fasting tests are more comfortable and convenient for individuals than fasting tests.^13^ In addition, fasting blood tests may be less safe for people with diabetes who can experience hypoglycemia when fasting.^22^, ^23^

Nonfasting triglyceride level was ~27 mg/dL higher compared to fasting samples in general,^22^, ^24^ which increment is believed to be not clinically significant for most individuals.^22^ Nonfasting samples seem to have at least the same prognostic value as fasting samples for general risk screening.^22^, ^25^ Some guidelines have started to recommend the use of non‐fasting triglyceride for general screening and risk evaluation. Examples of such guidelines include the American College of Cardiology/American Heart Association (ACC/AHA) guideline,^26^ the Canadian Cardiovascular Society Guideline,^23^ the European Society of Cardiology/European Atherosclerosis Society (ESC/EAS) guideline,^22^ and the British National Institute for Health and Care Excellence (NICE) guideline.^27^ The findings of the current study support the initiatives of these guidelines^22^, ^23^, ^26^, ^27^ to recommend the use of nonfasting triglyceride for general screening and risk evaluation.

The ACC/AHA guideline^26^ and the Canadian Cardiovascular Society Guideline^23^ further recommend that if a nonfasting triglycerides level is ≥400 mg/dL (≥4.5 mmol/L), a fasting test should be performed for assessment of fasting triglyceride levels. This current study does not support this further recommendation on repeating a fasting test, as a triglyceride level of ≥500 mg/dL was associated with an increased risk of all‐cause mortality in the nonfasting subcohort but not in the fasting subcohort.

This study found that, in the whole cohort comprising the fasting and nonfasting subcohorts, triglyceride was associated with all‐cause mortality. This result is consistent with two recent studies which reported similar findings.^9^, ^10^ The exact cause of death was not investigated by the current study; however, the result was consistent with previous observations that higher triglyceride was associated with higher mortality from cardiovascular disease^2^ and diabetes.^11^

This study found that fasting triglyceride was not associated with all‐cause mortality, which is consistent with some previous reports^4^, ^5^ but inconsistent with others.^1^, ^2^, ^3^ The lack of association in the current study is not due to a limitation in sample size, as this study included 27,036 participants with nonfasting triglyceride.

Why nonfasting triglyceride is more sensitive in predicting all‐cause mortality than its fasting counterpart is unknown. Out of a routine day with three meals (e.g., meals at 8 a.m., 12 noon, and 7 p.m., respectively), a person is in a fasting state for only about 5 h whereas in a non‐fasting state for the remaining 19 h. Therefore, non‐fasting triglyceride, which is about 27 mg/dL higher than the fasting value,^22^, ^24^ may more represent the impact of triglyceride on a person's health. It is worth noting that postprandial levels of triglyceride can be affected by many factors, for example, type of diet, time after a meal, and comorbidity. Therefore, an oral lipid tolerance test,^28^ similar to the oral glucose tolerance test, is expected to have higher clinical significance compared to a random nonfasting triglyceride test. This hypothesis needs to be investigated in the future.

This study found that the positive association between triglyceride and all‐cause mortality was presented in females but males. The underlying reason is unknown. The triglyceride‐related gender difference has been reported previously. For example, a meta‐analysis of 17 population‐based prospective studies showed that high triglyceride was a risk factor for cardiovascular disease in the general population,^29^ and the association seemed to be stronger in females than in males: relative risks (95% CIs) were 1.14 (1.05–1.28) and 1.37 (1.13–1.66) in males and females, respectively.^29^ Consistently, the Framingham Study showed that baseline triglyceride was positively associated with the incidence of coronary heart disease in females but not in males.^30^ Future studies are needed to investigate the reasons underlying the observed gender difference.

This study has a number of limitations. First, triglyceride was only measured at one timepoint, which may lead to misclassification. Second, this study was observational in nature and the causality could not be established.

## 5 CONCLUSION

This study demonstrated that fasting status modified the association between triglyceride and all‐cause mortality, and nonfasting triglyceride seemed better in predicting all‐cause mortality. This study supports the initiatives by some guidelines to suggest the use of nonfasting triglyceride for risk assessment.

## AUTHOR CONTRIBUTIONS

**Yan Fang**: Data curation; formal analysis; writing—original draft. **Yutang Wang**: Conceptualization; data curation; formal analysis; writing—original draft; writing—review & editing.

## CONFLICTS OF INTEREST

The authors declare that the research was conducted in the absence of any commercial or financial relationships that could be construed as a potential conflict of interest.

## TRANSPARENCY STATEMENT

Yutang Wang affirms that this manuscript is an honest, accurate, and transparent account of the study being reported with no important aspects of the study being omitted; and that any discrepancies from the study as planned have been explained.

## Supporting information

Supporting Information.

## Data Availability

The data that support the findings of this study are openly available on the NHANES website at https://www.cdc.gov/nchs/nhanes/index.htm.
