# Supplementary material for: Fasting status modifies the association between triglyceride and all‐cause mortality: A cohort study
Source: Health Sci Rep. 2022 May 16;5(3):e642. doi: 10.1002/hsr2.642 (PMC9110781; doi:10.1002/hsr2.642)
Supplement: Supplementary file 1 — Supporting Information. [file HSR2-5-e642-s001.docx]

**Table S1.** Baseline characteristics of the fasting sub-cohort

|  | Triglyceride concentration (mg/dL) | | | | |  | *P* for trend |
| --- | --- | --- | --- | --- | --- | --- | --- |
|  | < 100 | 100-149 | 150-199 | 200-499 | ≥ 500 | overall |  |
| Sample size | 11,519 | 7,444 | 3,819 | 3,926 | 328 | 27,036 | NA |
| Triglyceride, mg/dL, median (IQR) | 72  (59-85) | 121  (109-134) | 170  (159-183) | 251  (220-305) | 641  (551-824) | 110  (77-163) | < 0.001 |
| Age, y, mean (SD) | 45 (19) | 51 (19) | 52 (18) | 52 (17) | 49 (15) | 49 (19) | < 0.001 |
| Sex (male), n (%) | 5132 (44.6) | 3606 (48.4) | 1896 (49.6) | 2068 (52.7) | 232 (70.7) | 12934 (47.8) | < 0.001 |
| Ethnicity, n (%) |  |  |  |  |  |  | < 0.001 |
| Non-Hispanic white | 4698 (40.8) | 3475 (46.7) | 1871 (49.0) | 1981 (50.5) | 157 (47.9) | 12182 (45.1) |  |
| Non-Hispanic black | 3585 (31.1) | 1384 (18.6) | 515 (13.5) | 403 (10.3) | 28 (8.5) | 5915 (21.9) |  |
| Mexican American | 1964 (17.1) | 1750 (23.5) | 1009 (26.4) | 1142 (29.1) | 109 (33.2) | 5974 (22.1) |  |
| Other | 1272 (11.0) | 835 (11.2) | 424 (11.1) | 400 (10.2) | 34 (10.4) | 2965 (11.0) |  |
| Obesity, n (%) |  |  |  |  |  |  | < 0.001 |
| Underweight | 318 (2.8) | 90 (1.2) | 22 (0.6) | 17 (0.4) | 1 (0.3) | 448 (1.7) |  |
| Normal | 5010 (43.5) | 2131 (28.6) | 794 (20.8) | 619 (15.8) | 36 (11.0) | 8590 (31.8) |  |
| Overweight | 3487 (30.3) | 2659 (35.7) | 1449 (37.9) | 1511 (38.5) | 148 (45.1) | 9254 (34.2) |  |
| Obese | 2598 (22.6) | 2475 (33.2) | 1511 (39.6) | 1731 (44.1) | 141 (43.0) | 8456 (31.3) |  |
| Unknown | 106 (0.9) | 89 (1.2) | 43 (1.1) | 48 (1.2) | 2 (0.6) | 288 (1.1) |  |
| Poverty-income ratio, n (%) |  |  |  |  |  |  | < 0.001 |
| < 130% | 3208 (27.8) | 2096 (28.2) | 1111 (29.1) | 1226 (31.2) | 112 (34.1) | 7753 (28.7) |  |
| 130%-349% | 4208 (36.5) | 2840 (38.2) | 1389 (36.4) | 1428 (36.4) | 132 (40.2) | 9997 (37.0) |  |
| ≥ 350% | 3138 (27.2) | 1925 (25.9) | 949 (24.8) | 944 (24.0) | 62 (18.9) | 7018 (26.0) |  |
| Unknown | 965 (8.4) | 583 (7.8) | 370 (9.7) | 328 (8.4) | 22 (6.7) | 2268 (8.4) |  |
| Education, n (%) |  |  |  |  |  |  | < 0.001 |
| < High School | 3165 (27.5) | 2500 (33.6) | 1435 (37.6) | 1493 (38.0) | 129 (39.3) | 8722 (32.3) |  |
| High School | 2951 (25.6) | 1881 (25.3) | 978 (25.6) | 1024 (26.1) | 87 (26.5) | 6921 (25.6) |  |
| > High School | 5365 (46.6) | 3041 (40.9) | 1394 (36.5) | 1402 (35.7) | 112 (34.1) | 11314 (41.8) |  |
| Unknown | 38 (0.3) | 22 (0.3) | 12 (0.3) | 7 (0.2) | 0 (0) | 79 (0.3) |  |
| Physical activity, n (%) |  |  |  |  |  |  | < 0.001 |
| Inactive | 3445 (29.9) | 1807 (24.3) | 902 (23.6) | 879 (22.4) | 90 (27.4) | 7123 (26.3) |  |
| Insufficiently active | 4222 (36.7) | 2834 (38.1) | 1365 (35.7) | 1483 (37.8) | 112 (34.1) | 10016 (37.0) |  |
| Active | 3848 (33.4) | 2799 (37.6) | 1550 (40.6) | 1563 (39.8) | 126 (38.4) | 9886 (36.6) |  |
| Alcohol consumption, n (%) |  |  |  |  |  |  | < 0.001 |
| 0 drink/week | 1853 (16.1) | 1378 (18.5) | 736 (19.3) | 792 (20.2) | 62 (18.9) | 4821 (17.8) |  |
| < 1 drink/week | 2609 (22.6) | 1697 (22.8) | 871 (22.8) | 835 (21.3) | 59 (18.0) | 6071 (22.5) |  |
| 1-6 drinks/week | 2588 (22.5) | 1432 (19.2) | 677 (17.7) | 691 (17.6) | 65 (19.8) | 5453 (20.2) |  |
| ≥ 7 drinks/week | 1481 (12.9) | 941 (12.6) | 492 (12.9) | 501 (12.8) | 63 (19.2) | 3478 (12.9) |  |
| Unknown | 2988 (25.9) | 1996 (26.8) | 1043 (27.3) | 1107 (28.2) | 79 (24.1) | 7213 (26.7) |  |
| Smoking status, n (%) |  |  |  |  |  |  | < 0.001 |
| Past smoker | 2510 (21.8) | 1660 (22.3) | 898 (23.5) | 942 (24.0) | 92 (28.0) | 6102 (22.6) |  |
| Current smoker | 2436 (21.1) | 1962 (26.4) | 1123 (29.4) | 1169 (29.8) | 96 (29.3) | 6786 (25.1) |  |
| Non-smoker | 6564 (57.0) | 3818 (51.3) | 1795 (47.0) | 1813 (46.2) | 140 (42.7) | 14130 (52.3) |  |
| Hypertension, n (%) | 2846 (24.7) | 2553 (34.3) | 1401 (36.7) | 1549 (39.5) | 137 (41.8) | 8486 (31.4) | < 0.001 |
| Hypercholesterolemia, n (%) | 2000 (17.4) | 2087 (28.0) | 1255 (32.9) | 1469 (37.4) | 158 (48.2) | 6969 (25.8) | < 0.001 |
| Diabetes, n (%) | 658 (5.7) | 693 (9.3) | 437 (11.4) | 569 (14.5) | 75 (22.9) | 2432 (9.0) | < 0.001 |

Abbreviations: IQR, interquartile range; NA, not applicable; SD, standard deviation.

**Table S2.** Baseline characteristics of the non-fasting sub-cohort

|  | Triglyceride concentration (mg/dL) | | | | |  | *P* for trend |
| --- | --- | --- | --- | --- | --- | --- | --- |
|  | < 100 | 100-149 | 150-199 | 200-499 | ≥ 500 | overall |  |
| Sample size | 2,654 | 1,967 | 1,173 | 1,500 | 182 | 7,476 | NA |
| Triglyceride, mg/dL, median (IQR) | 75  (61-87) | 122  (110-135) | 171  (159-184) | 259  (224-319) | 609  (547-787) | 124  (84-189) | < 0.001 |
| Age, y, mean (SD) | 46 (19) | 51 (19) | 54 (19) | 54 (18) | 50 (15) | 50 (19) | < 0.001 |
| Sex (male), n (%) | 1097 (41.3) | 882 (44.8) | 560 (47.7) | 849 (56.6) | 121 (66.5) | 3509 (46.9) | < 0.001 |
| Ethnicity, n (%) |  |  |  |  |  |  | < 0.001 |
| Non-Hispanic white | 1091 (41.1) | 878 (44.6) | 555 (47.3) | 695 (46.3) | 79 (43.4) | 3298 (44.1) |  |
| Non-Hispanic black | 916 (34.5) | 517 (26.3) | 259 (22.1) | 231 (15.4) | 26 (14.3) | 1949 (26.1) |  |
| Mexican American | 497 (18.7) | 459 (23.3) | 290 (24.7) | 476 (31.7) | 69 (37.9) | 1791 (24.0) |  |
| Other | 150 (5.7) | 113 (5.7) | 69 (5.9) | 98 (6.5) | 8 (4.4) | 438 (5.9) |  |
| Obesity, n (%) |  |  |  |  |  |  | < 0.001 |
| Underweight | 122 (4.6) | 35 (1.8) | 5 (0.4) | 5 (0.3) | 0 (0) | 167 (2.2) |  |
| Normal | 1371 (51.7) | 674 (34.3) | 282 (24.0) | 292 (19.5) | 23 (12.6) | 2642 (35.3) |  |
| Overweight | 709 (26.7) | 720 (36.6) | 466 (39.7) | 632 (42.1) | 80 (44.0) | 2607 (34.9) |  |
| Obese | 438 (16.5) | 523 (26.6) | 407 (34.7) | 551 (36.7) | 78 (42.9) | 1997 (26.7) |  |
| Unknown | 14 (0.5) | 15 (0.8) | 13 (1.1) | 20 (1.3) | 1 (0.5) | 63 (0.8) |  |
| Poverty-income ratio, n (%) |  |  |  |  |  |  | 0.039 |
| < 130% | 761 (28.7) | 567 (28.8) | 362 (30.9) | 471 (31.4) | 64 (35.2) | 2225 (29.8) |  |
| 130%-349% | 1034 (39.0) | 757 (38.5) | 444 (37.9) | 571 (38.1) | 72 (39.6) | 2878 (38.5) |  |
| ≥ 350% | 630 (23.7) | 461 (23.4) | 252 (21.5) | 297 (19.8) | 29 (15.9) | 1669 (22.3) |  |
| Unknown | 229 (8.6) | 182 (9.3) | 115 (9.8) | 161 (10.7) | 17 (9.3) | 704 (9.4) |  |
| Education, n (%) |  |  |  |  |  |  | < 0.001 |
| < High School | 919 (34.6) | 762 (38.7) | 531 (45.3) | 723 (48.2) | 88 (48.4) | 3023 (40.4) |  |
| High School | 778 (29.3) | 589 (29.9) | 327 (27.9) | 401 (26.7) | 56 (30.8) | 2151 (28.8) |  |
| > High School | 942 (35.5) | 600 (30.5) | 304 (25.9) | 368 (24.5) | 37 (20.3) | 2251 (30.1) |  |
| Unknown | 15 (0.6) | 16 (0.8) | 11 (0.9) | 8 (0.5) | 1 (0.5) | 51 (0.7) |  |
| Physical activity, n (%) |  |  |  |  |  |  | < 0.001 |
| Inactive | 927 (34.9) | 649 (33.0) | 347 (29.6) | 446 (29.7) | 54 (29.7) | 2423 (32.4) |  |
| Insufficiently active | 1043 (39.3) | 760 (38.6) | 458 (39.0) | 562 (37.5) | 71 (39.0) | 2894 (38.7) |  |
| Active | 684 (25.8) | 558 (28.4) | 368 (31.4) | 492 (32.8) | 57 (31.3) | 2159 (28.9) |  |
| Alcohol consumption, n (%) |  |  |  |  |  |  | < 0.001 |
| 0 drink/week | 446 (16.8) | 377 (19.2) | 230 (19.6) | 304 (20.3) | 35 (19.2) | 1392 (18.6) |  |
| < 1 drink/week | 322 (12.1) | 251 (12.8) | 148 (12.6) | 185 (12.3) | 11 (6.0) | 917 (12.3) |  |
| 1-6 drinks/week | 543 (20.5) | 357 (18.1) | 179 (15.3) | 206 (13.7) | 28 (15.4) | 1313 (17.6) |  |
| ≥ 7 drinks/week | 321 (12.1) | 241 (12.3) | 128 (10.9) | 185 (12.3) | 28 (15.4) | 903 (12.1) |  |
| Unknown | 1022 (38.5) | 741 (37.7) | 488 (41.6) | 620 (41.3) | 80 (44) | 2951 (39.5) |  |
| Smoking status, n (%) |  |  |  |  |  |  | < 0.001 |
| Past smoker | 681 (25.7) | 501 (25.5) | 306 (26.1) | 373 (24.9) | 58 (31.9) | 1919 (25.7) |  |
| Current smoker | 548 (20.6) | 507 (25.8) | 340 (29.0) | 469 (31.3) | 65 (35.7) | 1929 (25.8) |  |
| Non-smoker | 1425 (53.7) | 958 (48.7) | 527 (44.9) | 658 (43.9) | 59 (32.4) | 3627 (48.5) |  |
| Hypertension, n (%) | 575 (21.7) | 651 (33.1) | 426 (36.3) | 613 (40.9) | 71 (39.0) | 2336 (31.2) | < 0.001 |
| Hypercholesterolemia, n (%) | 327 (12.3) | 382 (19.4) | 304 (25.9) | 471 (31.4) | 72 (39.6) | 1556 (20.8) | < 0.001 |
| Diabetes, n (%) | 183 (6.9) | 219 (11.1) | 186 (15.9) | 329 (21.9) | 56 (30.8) | 973 (13.0) | < 0.001 |

Abbreviations: IQR, interquartile range; NA, not applicable; SD, standard deviation.

**Table S3.** Interaction of triglyceride with sex in predicting all-cause mortality analyzed by Cox proportional hazards models

|  | HR † | 95% CI | *P* value |
| --- | --- | --- | --- |
| LnTG X Sex ‡ | 1.15 | 1.07-1.25 | <0.001 |

Abbreviations: CI, confidence interval; HR, hazard ratio; LnTG, natural log-transformed triglyceride.

† Adjusted for triglyceride (natural log-transformed), fasting status, age, sex, ethnicity, obesity, poverty-income ratio, education, physical activity, alcohol consumption, smoking status, survey period, hypercholesterolemia, hypertension, diabetes, and interaction between triglyceride and sex (LnTG X Sex).

‡ The interaction factor, computed as natural log-transformed triglyceride multiplied by sex (male and female, coded as 1 and 2, respectively). The resulting interaction factor was treated as a continuous variable in the interaction analysis.

**Table S4.** Natural log-transformed triglyceride and risk for all-cause mortality among 34,512 adults, stratified by sex

| Whole cohort  (N = 34,512) | | | Male  (N = 16,443) | | | Female  (N = 18,069) | | |
| --- | --- | --- | --- | --- | --- | --- | --- | --- |
| HR † | 95% CI | *P* value | HR † | 95% CI | *P* value | HR † | 95% CI | *P* value |
| 1.08 | 1.04-1.13 | <0.001 | 1.03 | 0.98-1.09 | 0.26 | 1.16 | 1.09-1.24 | <0.001 |

Abbreviations: CI, confidence interval; HR, hazard ratio.

† Adjusted for triglyceride (natural log-transformed), fasting status, age, sex, ethnicity, obesity, poverty-income ratio, education, physical activity, alcohol consumption, smoking status, survey period, hypercholesterolemia, hypertension, and diabetes.

**Table S5.** Interaction of triglyceride with ethnicity in predicting all-cause mortality analyzed by Cox proportional hazards models

|  | HR † | 95% CI | *P* value |
| --- | --- | --- | --- |
| LnTG X Ethnicity ‡ | 0.97 | 0.92-1.01 | 0.12 |

Abbreviations: CI, confidence interval; HR, hazard ratio; LnTG, natural log-transformed triglyceride.

† Adjusted for triglyceride (natural log-transformed), fasting status, age, sex, ethnicity, obesity, poverty-income ratio, education, physical activity, alcohol consumption, smoking status, survey period, hypercholesterolemia, hypertension, diabetes, and interaction between triglyceride and ethnicity (LnTG X Ethnicity).

‡ The interaction factor, computed as natural log-transformed triglyceride multiplied by ethnicity (non-Hispanic white, non-Hispanic black, Mexican-American, or other, coded as 1, 2, 3, and 4, respectively). The resulting interaction factor was treated as a continuous variable in the interaction analysis.

**Table S6.** Sensitivity analysis of the association between natural log-transformed triglyceride and risk for all-cause mortality among 34,512 adults

|  | Whole cohort  (N = 34,512) | | | Fasting sub-cohort  (N = 27,036) | | | Non-fasting sub-cohort (N = 7,476) | | |
| --- | --- | --- | --- | --- | --- | --- | --- | --- | --- |
|  | HR | 95% CI | *P* value | HR | 95% CI | *P* value | HR | 95% CI | *P* value |
| Model 1 | 1.08 | 1.04-1.13 | <0.001 | 1.05 | 0.99-1.10 | 0.11 | 1.17 | 1.09-1.25 | <0.001 |
| Model 2 | 1.07 | 1.03-1.12 | 0.001 | 1.04 | 0.98-1.09 | 0.17 | 1.15 | 1.07-1.23 | <0.001 |
| Model 3 | 1.07 | 1.03-1.12 | 0.001 | 1.04 | 0.98-1.09 | 0.17 | 1.14 | 1.06-1.22 | <0.001 |

Abbreviations: CI, confidence interval; HR, hazard ratio.

Model 1: adjusted for age, sex, ethnicity, obesity, poverty-income ratio, education, physical activity, alcohol consumption, smoking status, survey period, hypercholesterolemia, hypertension, and diabetes.

Model 2: adjusted for all the factors in Model 1 plus history of heart attack (yes, no, or unknown).

Model 3: adjusted for all the factors in Model 2 plus history of stroke (yes, no, or unknown).
